# Risk factors and prediction model of delirium in elderly patients after hip arthroplasty

**DOI:** 10.12669/pjms.40.6.9306

**Published:** 2024-07

**Authors:** Yanli Duan, Ruzhen Zhang

**Affiliations:** 1 Yanli Duan, Department of Orthopedics and Joints, Wuhan Fourth Hospital, Wuhan 430000, P.R. China; 2 Ruzhen Zhang, Department of Traumatology and Orthopedics, Wuhan Fourth Hospital, Wuhan 430000, P.R. China

**Keywords:** Hip arthroplasty, Delirium, Risk factors, Prediction model, Nomogram

## Abstract

**Objective:**

To analyze the risk factors of delirium in elderly patients after hip arthroplasty and to construct a prediction model.

**Methods:**

Clinical data of 248 elderly patients who underwent hip arthroplasty in the Department of Traumatology and Orthopedics at Wuhan Fourth Hospital were retrospectively collected from November 2021 to February 2023. Logistic regression analysis was used to identify the risk factors of delirium after hip arthroplasty, and a nomogram prediction model was constructed using the RMS package of R4.1.2 software. The accuracy and stability of the model was evaluated based on the Hosmer-Lemeshow goodness-of-fit test and the receiver operating characteristic (ROC) curve.

**Results:**

Age, nighttime sleep, anesthesia method, intraoperative blood loss, hypoxemia, and C-reactive protein (CRP) level were all risk factors of delirium after the hip arthroplasty (P<0.05). These factors were used to construct a nomogram prediction model that was internally validated using the Bootstrap method. The prediction model had the area under ROC curve (AUC) of 0.980 (95% CI: 0.964-0.996), indicating that it has certain predictive value for postoperative delirium. When the optimal cut off value was selected, the sensitivity and specificity were 92.7% and 92.3%, respectively, indicating that the prediction model is effective.

**Conclusions:**

Age, short nighttime sleep, general anesthesia, high intraoperative blood loss, hypoxemia, and high CRP levels are independent risk factors for delirium after hip arthroplasty. The nomogram prediction model constructed based on these risk factors can effectively predict delirium in elderly patients after hip arthroplasty.

## INTRODUCTION

With a gradual aging of the population worldwide, risks of hip joint-related diseases such as femoral head necrosis, osteoarthritis, and hip fractures among the elderly population are also on the rise, posing a serious problem in this group of patients.^1^,^2^ Hip arthroplasty, performed more than one million each year worldwide, is the main method for clinical treatment of hip joint-related diseases, that allows to restore limb function and improve the quality of life.^3^–^5^ However, elderly population of patients generally presents with an overall lower tolerance to invasive surgical procedures and anesthesia,^6^ with higher risk of postoperative complications, including delirium.^6^–^8^ Postoperative delirium often occurs 2-5 days after the surgery, and its incidence can reach 50% to 70% in high-risk populations.^9^ It can significantly prolong the hospitalization period, increasing the incidence of deep vein thrombosis, electrolyte disorders, lung infections, etc.^10^,^11^ Studies have shown that patients with delirium after fracture surgery have about three times higher risk of mortality after the procedure compared to patients without delirium.^11^,^12^ Therefore, it is important to identify the potential risk factors of delirium after hip arthroplasty, especially in the elderly population of patients.

At present, studies have investigated risk factors for postoperative delirium in elderly patients after hip arthroplasty, however, the predictive efficiency of the models varies.^13^-^15^ This study aimed to identify risk factors of delirium after hip arthroplasty and to construct a prediction model to validate the findings of previous studies, which may contribute to develop strategies to prevent delirium after hip arthroplasty through early intervention, thus improving the quality of life of elderly patients.

## METHODS

Records of 248 elderly patients who underwent hip arthroplasty in the Department of Traumatology and Orthopedics at Wuhan Fourth Hospital from November 2021 to February 2023 were collected. Of them, 53 patients with postoperative delirium were assigned to the delirium group, and 195 patients without postoperative delirium were assigned to the non-delirium group.

### Ethical Approval

The ethics committee of Wuhan Fourth Hospital approved this study (August 5^th^ 2023, No. KY2023-080-01).

### Inclusion criteria:

- Patients had surgical indications and underwent hip arthroplasty for the first time.
- Age ≥ 60 years old.
- The clinical data was complete with follow up of at least six months.
- The American Society of Anesthesiologists (ASA) classification of Grade I-III.

### Exclusion criteria:

- Patients with preoperative mental diseases or cognitive dysfunction.
- Previous history of brain surgery.
- Individuals with Parkinson’s disease, dementia, traumatic brain injury, and stroke.
- Patients complicated with other fractures.

### Outcome measures

Gender, hypertension, diabetes, chronic obstructive pulmonary disease (COPD), body mass index (BMI), age, night sleep time, anesthesia mode, intraoperative blood loss, hypoxemia, and CRP level.

### Postoperative delirium

Diagnosis of delirium was made according to the Diagnostic and Statistical Manual of Mental Disorders (5^th^ edition)^16^ and evaluated using the Confusion Assessment Method-Chinese Reversion (CAM-CR).^17^ The CAM-CR scale involves 11 items, including acute disease course, attention deficit, cognitive disorder, consciousness disorder, memory loss, disorientation, perceptual disorder, excitation delay, disease fluctuation, and changes in sleep and wakefulness cycles. A score of 1-4 indicates non-existent, mild, moderate, and severe delirium, with a score range of 11-44 points. Score is 22 or above indicates the presence of delirium.^18^ Higher score indicates more severe postoperative delirium.

### Statistical analysis

The statistical software used were SPSS22.0 and the R software version 4.0.0. The measurement data conforming to a normal distribution was represented by (*χ̅*±*S*), while independent sample t-tests was used for inter group comparisons. The econometric data conforming to an abnormal distribution was represented by M (IQR), with Mann Whitney U test used for inter group comparison. Counting data was represented by n (%), and inter group comparisons were made using *χ^2^* tests. Logistic regression models were used to analyze the risk factors of delirium. Based on the identified risk factors, a nomogram prediction model was constructed using the “rms” package in R software. The accuracy and stability of the model was evaluated based on the Hosmer-Lemeshow goodness-of-fit test and the receiver operating characteristic (ROC) curve. P<0.05 was considered statistically significant.

## RESULTS

A total of 248 elderly hip arthroplasty patients were included in this study. Among them, 53 patients experienced postoperative delirium. There were significant differences between the two groups in age, nighttime sleep time, anesthesia methods, intraoperative blood loss, hypoxemia, and CRP levels (*P*<0.05), Table-I.

**Table-I:** Comparison of General Conditions between Two Groups.

Index	Non-delirium group (n=195)	Delirium group (n=53)	t/χ^2^	P
Age (year)	72.27±6.74	77.85±5.95	-5.476	<0.001
Male [n (%)]	98(50.26)	32(60.38)	1.712	0.191
BMI (kg/m^2^)	24.97±2.17	24.81±2.21	0.480	0.632
Hypertension [yes, n (%)]	107(54.87)	31(58.49)	0.221	0.638
Diabetes [yes, n (%)]	111(56.92)	34(64.15)	0.897	0.344
COPD [yes, n (%)]	93(47.69)	26(49.06)	0.031	0.860
Night Sleep Time (hours)	5(4,6)	7(6,9)	-6.391	<0.001
Intraoperative blood loss (ml)	250(220,280)	350(310,390)	-8.270	<0.001
General anesthesia [n (%)]	137(70.26)	49(92.45)	10.950	0.001
Hypoxemia [yes, n (%)]	21(10.77)	16(30.19)	12.381	<0.001
CRP (mg/L)	38.00±7.53	49.02±7.80	-9.373	<0.001

Age, nighttime sleep time, anesthesia method, intraoperative blood loss, hypoxemia, and CRP level were all independent risk factors for delirium after hip arthroplasty (P<0.05), Table-II.

**Table-II:** Risk factors related to delirium after THA.

Variable	B	S.E.	Wald	P	OR	95%CI
Older	0.319	0.079	16.301	<0.001	1.375	1.178-1.605
Short sleep time at night	0.837	0.213	15.395	<0.001	2.310	1.520-3.509
General anesthesia	2.018	0.823	6.019	0.014	7.526	1.501-37.740
Hypoxemia	1.939	0.830	5.460	0.019	6.949	1.367-35.333
Large intraoperative blood loss	0.032	0.008	17.719	<0.001	1.033	1.018-1.049
High CRP level	0.219	0.059	13.77	<0.001	1.244	1.109-1.397

**Fig.1:**
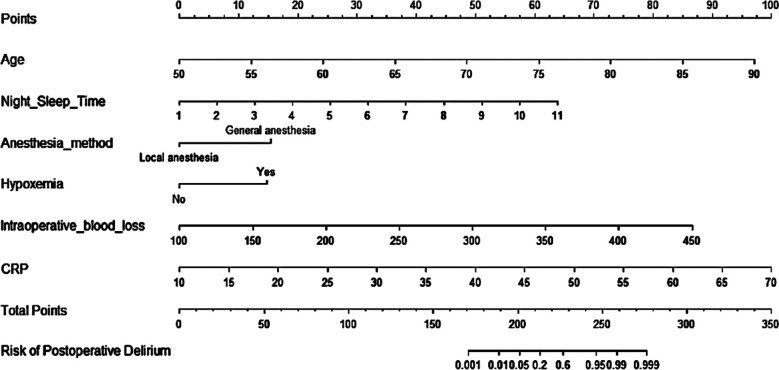
Nomogram Prediction Model.

**Fig.2:**
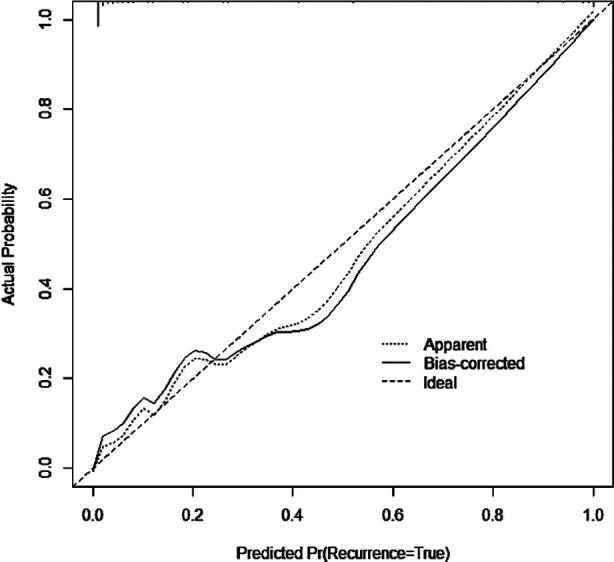
Calibration Curve.

A nomogram prediction model of meaningful risk factors was done by a multivariate logistic regression model, including age, nighttime sleep time, anesthesia method, hypoxemia, intraoperative blood loss, and CRP. The nomogram model predicted postoperative delirium in patients, and the C-index of the nomogram prediction model was 0.980, reflecting a good potential clinical efficacy of the prediction model, Fig.1. The calibration curve showed good consistency between actual observations and nomogram predictions, Fig.2. According to the nomogram prediction model, the prediction model equation was: total score=2.433 * age+6.382 * night sleep time+15 * anesthesia method+15 * hypoxemia+0.248 * intraoperative blood loss+1.668 * CRP-169.861. ROC curve was established to analyze the value of the model in predicting postoperative delirium. The results showed that the AUC (95% CI) of the model was 0.980 (0.964, 0.996), indicating a high predictive value for postoperative delirium. When the optimal cut off value was selected, the sensitivity and specificity were 92.7% and 92.3%, respectively, indicating that the prediction model is effective.

**Fig.3:**
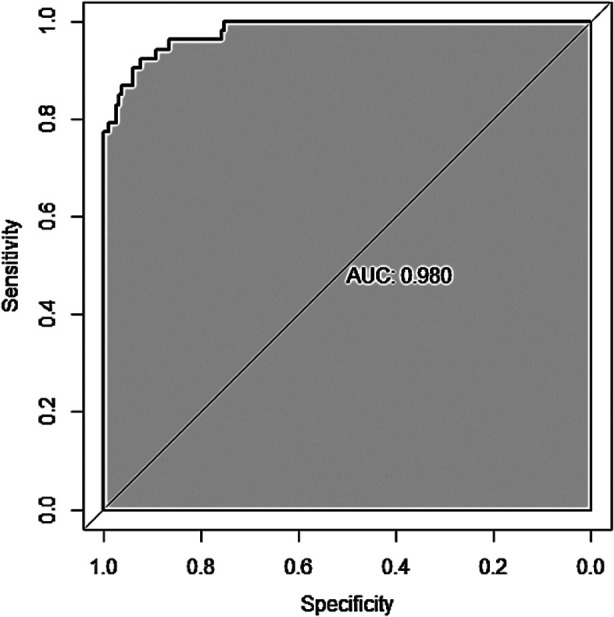
ROC curve.

## DISCUSSION

The results of this study indicated that the incidence of delirium in elderly patients after hip arthroplasty was 21.37% (53/248). Previous reports demonstrated a certain variability in the rate of postoperative delirium in elderly orthopedic patients. Elderly patients with total hip arthroplasty are considered one of the high-risk groups for postoperative delirium, with the incidence ranging from 4% to 53%.^19^ Gao et al.^20^ analyzed data of 365 elderly patients undergoing hip arthroplasty, and found that the incidence of postoperative delirium was 26%. Additionally, age, frailty, and high CRP levels were all identified as risk factors for postoperative delirium. Lu et al.^21^ showed that the incidence of postoperative delirium in elderly patients with total hip arthroplasty was as high as 40.5%, and age and stroke history were all identified as risk factors for delirium. The incidence of postoperative delirium in our study is within the range, defined in the previous studies, further strengthening the need to explore strategies to reduce the incidence of this adverse effect of the surgery.

Our study found that older age, shorter nighttime sleep, general anesthesia, high intraoperative blood loss, hypoxemia, and high CRP levels are all important risk factors for delirium after hip arthroplasty. We may speculate that compared to young patients, older individuals experience varying degrees of organ dysfunction, with degenerative changes in their brain tissue and a gradual decline in central nervous system function. Multiple organ functions and compensatory abilities also decrease, making the patients prone to cerebral ischemia. In addition, postoperative abnormalities in brain biochemical metabolism and physiological function may increase the risk of delirium.^20^,^21^

In terms of the sleep time, elderly patients undergoing hip joint surgery are highly susceptible to pain caused by invasive surgical procedures, medical activities, noise, environmental changes, and other related factors, resulting in reduced sleep time and decreased sleep quality. Usually, benzodiazepines or other hypnotic and sedative drugs are required, which can easily cause delirium. Zhou et al.^22^ found that the use of hypnotic and sedative drugs is an important factor leading to delirium in elderly patients after knee joint and hip arthroplasty.

Anesthesia method was identified as another risk factor in our study. Compared to local anesthesia, general anesthesia requires the use of various anesthetic analgesics, anticholinergic drugs, sedative and hypnotic drugs, which increase the risk of postoperative delirium.^23^ Midazolam, a benzodiazepine type of general anesthesia drug, can have varying degrees of inhibitory effects on the body’s circulation, especially in elderly patients, which can easily lead to a decrease in their responsiveness to environmental stimuli.^23^,^24^

Contrary to the results of Guo et al^13^ and Chen et al^25^, we found that advanced age is an independent risk factor for delirium in elderly patients after hip arthroplasty. We speculate that excessive blood loss during hip joint surgery can have varying degrees of impact on the stability of blood circulation, thereby affecting tissue oxygen supply and blood supply status, blood pressure stability, causing brain tissue hypoxia, ischemia, and triggering delirium.^26^

Our study showed that hypoxemia was a risk factor for postoperative delirium in elderly patients. A degenerative change in organ function in elderly patients undergoing hip joint surgery may lead to reduced sensitivity of the body’s blood pressure regulation mechanism. Anesthesia may cause a low blood pressure, and subsequent decrease in cerebral perfusion, leading to ischemia and hypoxia of brain tissue, abnormal brain metabolism and weakened function, and delirium related symptoms.^27^

Serum levels of CRP are relatively low under normal physiological conditions.^28^ Abnormally high serum expression of CRP can damage the blood-brain barrier and promote the production of large quantities of inflammatory factors by glial cells, triggering neurotoxic reactions and increasing the risk of delirium. Uzoigwe et al.^28^ also showed that postoperative delirium and cognitive decline are common types of complications after hip fracture surgery, and preoperative psychological state test scores and ASA grading are closely related to postoperative delirium. It can serve as an important factor in predicting postoperative delirium, thereby identifying high-risk individuals for postoperative delirium early and providing corresponding interventions in a timely manner.

Nomogram has been widely used for predicting postoperative events after hip arthroplasty.^29^-^31^ We used the identified risk factors to construct and validate a risk prediction model for delirium after hip arthroplasty. Our study shows that this model can achieve good prediction results, which has a higher AUC (0.980) than studies by Yang et al^15^ and Chen et al^25^, but similar to Li et al.^32^ Therefore, we suggest referring to the model to predict the risk of postoperative delirium in elderly patients after hip arthroplasty. Our prediction model may allow to further strengthen perioperative management, including perioperative health education and psychological intervention, and to reduce the occurrence of postoperative complications, promote the smooth recovery of hip joint function, and achieve effective prevention of postoperative delirium.

### Limitations

This is a retrospective study with some recall bias. A small sample size and fewer cases in the postoperative delirium group may result in a weak correlation between the results and the study factors. Additionally, this study did not explore the correlation between preoperative psychological status, ASA grading, and postoperative delirium, and further research is needed to confirm our results.

## CONCLUSION

Age, short nighttime sleep, general anesthesia, high intraoperative blood loss, hypoxemia, and high CRP levels are all independent risk factors for delirium after hip arthroplasty. The nomogram prediction model constructed based on these risk factors can effectively predict delirium in elderly patients after hip arthroplasty.

### Authors’ contributions:

**YD** conceived and designed the study.

**YD and RZ** collected the data and performed the analysis.

**YD** was involved in the writing of the manuscript and is responsible for the integrity of the study.

All authors have read and approved the final manuscript.
